# Nephroprotective effects of enalapril after [177Lu]-DOTATATE therapy using serial renal scintigraphies in a murine model of radiation-induced nephropathy

**DOI:** 10.1186/s13550-016-0219-2

**Published:** 2016-08-11

**Authors:** Harun Ilhan, Hao Wang, Franz J. Gildehaus, Carmen Wängler, Tanja Herrler, Andrei Todica, Julia Schlichtiger, Paul Cumming, Peter Bartenstein, Marcus Hacker, Alexander R. Haug

**Affiliations:** 1 Department of Nuclear Medicine, University of Munich, Munich, Germany; 2 Department of Surgery, Campus Großhadern, University of Munich, Munich, Germany; 3 Department of Biomedical Imaging and Image-Guided Therapy, Division of Nuclear Medicine, University of Vienna, Vienna, Austria; 4 Institute of Pharmacy and Molecular Biotechnology, University of Heidelberg, Heidelberg, Germany

**Keywords:** [177Lu]-DOTATATE, Radiation-induced nephropathy, Enalapril, Tc-99m-MAG3 scintigraphy, Neuroendocrine tumors

## Abstract

**Background:**

Radiation-induced nephropathy is still dose limiting in radionuclide therapy of neuroendocrine tumors. We investigated the nephroprotective potential of the angiotensine converting enzyme inhibiting drug enalpril after [177Lu]-DOTATATE therapy in a murine model of radiation-induced nephropathy by renal scintigraphy.

At first, the appropriate therapy activity to induce nephropathy was identified. Baseline scintigraphy (*n* = 12) entailed 12-min dynamic acquisitions after injection of 25 MBq [99mTc]-MAG3, which was followed by radionuclide therapy at four escalating activities of [177Lu]-DOTATATE: group (Gp) 1: 10 MBq; Gp 2: 20 MBq; Gp 3: 40 MBq; Gp 4: 65 MBq. Follow-up [99mTc]-MAG3 scintigraphy was carried out at days 9, 23, 44, and 65. The treatment activity for the intervention arm was selected on the basis of histological examination and declining renal function. In the second part, daily administration by gavage of 10 mg/kg/d enalapril or water (control group) was initiated on the day of radionuclide therapy. Follow-up scintigraphy was carried out at days 9, 23, 44, 65, and 86. We also created a non-therapy control group to detect therapy-independent changes of renal function over time. For all scintigraphies, mean renogram curves were analyzed and the “fractional uptake rate” (FUR; %I.D./min ± SEM) of the tracer by the kidneys was calculated as an index of renal clearance.

**Results:**

At day 65 of follow-up, no significant change in the FUR relative to baseline (11.0 ± 0.3) was evident in radionuclide therapy groups 1 (11.2 ± 0.5) and 2 (10.1 ± 0.6), but FUR was significantly reduced in groups 3 (8.93 ± 0.6, *p* < 0.05) and 4 (6.0 ± 0.8, *p* < 0.01); we chose 40 MBq [177Lu]-DOTATATE (Gp 3) for the intervention study. Here, at the last day of follow-up (day 86), FUR was unaltered in enalapril-treated mice (11.8 ± 0.5) relative to the baseline group (12.4 ± 0.3) and non-therapy group (11.9 ± 0.8), whereas FUR in the control group had undergone a significant decline (9.3 ± 0.5; *p* < 0.01). Histological examination revealed prevention of kidney damage by enalapril treatment.

**Conclusions:**

Treatment with enalapril is effective for nephroprotection during radionuclide therapy with [177Lu]-DOTATATE in mice. Although these results are only limitedly transferable to human studies, enalapril might serve as a promising drug in the mitigation of nephropathy following treatment with [177Lu]-DOTATATE.

**Electronic supplementary material:**

The online version of this article (doi:10.1186/s13550-016-0219-2) contains supplementary material, which is available to authorized users.

## Background

Peptide receptor radionuclide therapy (PRRT) with [Y-90-DOTA^0^,TYR^3^]-octreotide ([90Y]-DOTATOC) and [177Lu-DOTA^0^,TYR^3^]-octreotate ([177Lu]-DOTATATE) has emerged as a well-established therapy option in the treatment of inoperable or metastasized neuroendocrine tumors (NET) expressing somatostatin receptors [1, 2]. As reported in several studies, PRRT yields encouraging results with respect to favorable response rates and progression-free survival [1–8]. However, an important factor limiting benefits of this therapy is the occurrence of radiation-induced nephropathy, which arises due to renal clearance and megalin-receptor mediated tubular reabsorption of the chelated somatostatin analogues [9, 10]. Significant impairment in renal function has been reported both in clinical and in pre-clinical surveys [11–17]. There are various pharmacological strategies to decrease renal radiation exposure, for instance inhibiting tubular reabsorption. The co-administration of positively charged amino-acids, mainly arginine and lysine, reduces renal uptake of radiolabeled somatostatin analogues both in animal studies [16, 18] and in patient studies [19, 20]. Histopathological findings in kidneys after PRRT present a pattern of tubular and glomerular damage which seems similar to that evoked by external beam radiation [21].

The renin-angiotensin-aldosterone-system (RAAS) is also decisively implicated in the pathogenesis of radiation-induced nephropathy [21, 22]. RAAS-modifying pharmacotherapies have been ascertained for the reduction of renal damage after radiation in animal models. The nephroprotective potential of angiotensin-converting-enzyme (ACE) inhibitors after external beam radiation of the kidneys in rats was first reported by Cohen et al. in 1992 [23]. The mitigative effects of ACE inhibitors were confirmed in further pre-clinical experiments by the same and other research groups [24, 25], as well as in humans by Moll et al. [12].

Given the similar pathologies and histopathological changes, we hypothesized that the pharmacological treatments effective in nephrotoxity induced by external beam radiation might also mitigate [177Lu]-DOTATATE induced nephrotoxicity.

## Methods

### Animals and experimental design

All animal experiments were conducted in accordance with institutional guidelines and approved by the ethics committee and Administrative Panel on Laboratory Animal Care (Government of Upper Bavaria, Germany; reference number 55.2-1-54-2531-136-09). We used female Balb C mice, aged 10 weeks and weighing 20 to 24 g (Charles River Laboratories, Sulzfeld, Germany), which were fed a standard diet and given free access to water.

No-carrier added lutetium-177 was obtained from Isotope Technologies Garching GmbH (Garching, Germany). DOTA^0^,TYR^3^-octreotate was obtained from ABX advanced biochemical compounds (Dresden, Germany). Radiolabeling was performed according to a previously described protocol [26, 27]. Other pharmaceuticals were obtained from Sigma-Aldrich (Taufkirchen, Germany).

The study consisted of two parts. In the first part, we investigated the activity-dependent impairment of renal function in mice treated with four activities of [177Lu]-DOTATATE: group 1 (*n* = 6), 10 MBq; group 2 (*n* = 8), 20 MBq; group 3 (*n* = 7), 40 MBq, and group 4 (*n* = 7), 65 MBq. PRRT was carried out at day 2. [177Lu]-DOTATATE was injected via the tail vein. Follow-up scintigraphies to assess renal function were carried out at days 9, 23, 44, and 65.

In the second part of the study, the nephroprotective properties of enalapril were evaluated in mice (*n* = 7) treated with 40 MBq [177Lu]-DOTATATE, as selected in part one. PRRT was performed under anesthesia as described above. Enalapril was administered by gavage on the day of radionuclide therapy, and daily thereafter until the end of the study. The pharmaceutical dose (10 mg/kg) was chosen in accordance with previously published studies [28]. Control mice (*n* = 7) received daily gavage with sterile water. Furthermore, we created a third group of (*n* = 7) mice, which served as a non-therapy control. In this group, no PRRT was carried out and mice received MAG3 scintigraphy to identify therapy-independent changes in renal function over 3 months in healthy mice.

MAG3 scintigraphy was obtained at baseline in order to determine the renal function prior to PRRT. Follow-up renal scintigraphies to assess renal function were carried out in all mice at days 9, 23, 44, 65, and 86.

### Renal scintigraphy

MAG3 scintigraphy (Technescan MAG3, Covidien, Neustadt/Donau, Germany) was performed as previously described [29–31]. After the induction of anesthesia with a combination of ketamine (75 mg/kg, i.p.) and medetomidine (1 mg/kg, i.p.), mice were placed on a thermostatically heated pad to maintain body temperature close to 37 °C. Mice received a standard activity of 25 MBq [99mTc]-MAG3 administered in 150 μl saline as a bolus via a tail vein. Whole body scintigraphic recordings using one head of a triple-headed gamma camera (Philips—former Picker—Prism 3000 XP, Cleveland, USA) equipped with a LEHR collimator were initiated upon tracer administration. The dynamic planar acquisitions consisted of 144 frames of 5 s each, to a total of 12 min. The image magnification was set to four times.

**Fig. 1:**
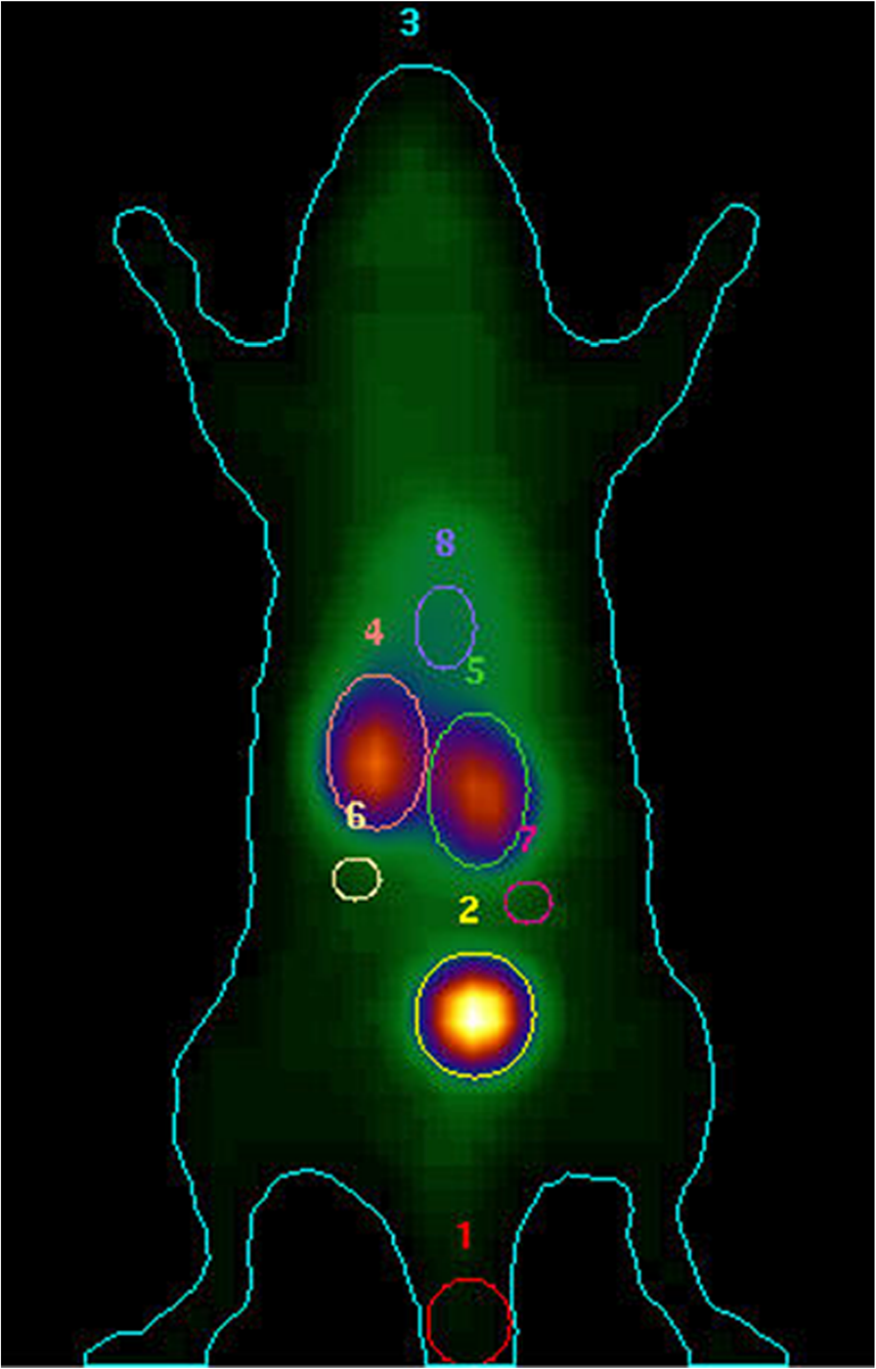
Image file at 12-min examination. Time activity curves were obtained by standard manual region of interest (ROI) analyses of the whole body (3), kidneys (4 and 5), kidney backgrounds (6 and 7), and site of injection (1)

Image files were analyzed using Hermes Dynamic Study display software V4.0 (Hermes Gold V2.10, Hermes Medical Solutions, Stockholm/London). Standard region of interest (ROI) were applied to the whole body, both kidneys, peri-renal background reference regions, bladder, blood pool in the heart, and the site of injection (Fig. 1) [31]. The dynamic data for each ROI were exported to Microsoft Excel to evaluate renal function, which was represented as renograms depicting percentage of injected activity (%IA). The fractional uptake rate (FUR), which constitutes a measure of renal clearance, was calculated as described previously [30]. In brief, the FUR is defined as the fractional uptake of a tracer in the blood by an organ as a function of time. FUR is calculated as FUR = *P*(0)*(*k*_l_ + *k*_r_)/[ID], where *P*(0) (counts per second) is the magnitude of the plasma clearance curve *P*(*t*) at time zero, which is obtained by back extrapolation using a mono-exponential fitting of the data to *P*(*t*), and *k*_l_ and *k*_r_ are the slopes of the linear uptake (LU) segment of the Patlak-Rutland (PR) plots for the left and right kidneys [32].

### Histopathological analysis

At the end of the 3-month follow-up, mice were euthanized by cervical dislocation. Kidneys were removed, fixed in 4 % formalin, and embedded in paraffin. Histological sections were cut on a microtome and stained with hematoxylin-eosin (H.E.) and periodic acid-Schiff reagent (PAS). Renal damage was evaluated qualitatively according to a previously described scale [16, 17], ranging from no histological damage to severe histological damage based on htethe assessment criteria listed in Table 1. Whenever one of the criteria (e.g., tubular dilatation, cell-rich infiltrate) was evident, the damage was scored in the respective highest damage score.

**Table 1:** Histological assessment criteria for renal damage (adapted from Rolleman et. al, EJNMMI 2007 [16])

Grade	Overview	Glomeruli	Tubules
Mild	More or less normal aspect	Apoptosis of endothelium cells	Apoptotic cells
	High glomerular cell count	Inflammatory infiltrate	Rough protein staining
			Little dilation
			Normal basal membrane
			No proteine cylinders
Moderate	Tubular dilation	Same as grade 1	More apoptotic cells
	Tubular cell damage		More pronounced dilation
			Thickened basal membrane
			Little tubular protein cylinders
			Regenerating cells (mitotic activity)
Pronounced	Stronger tubular dilation	Smaller vascular lumina, few erythrocytes	Flat epithelium
	Cell-rich infiltrate		Partly, complete loss of epithelium
	Regenerating tubules		Stronger dilation
			Inflammatory infiltrate
			Regeneration present
			More thickened basal membrane
Severe	Severe tubular dilation	Same as grade 3	Same as grade 3, but more empty cylinders
		More optical empty space due to glomerular shrinkage	Peripheral fibrosis

### Statistical analysis

Data are expressed as mean ± SEM. Normality was tested with the Shapiro-Wilk test. The paired or unpaired *t* test (two-sided) was used to test statistical significant differences of normally distributed results, and the Mann-Whitney-*U*-test was used when normality requirements were not met, with *p* < 0.05 considered in both cases as statistically significant.

## Results

### Identification of nephrotoxic activity of [177Lu]-DOTATATE

**Fig. 2:**
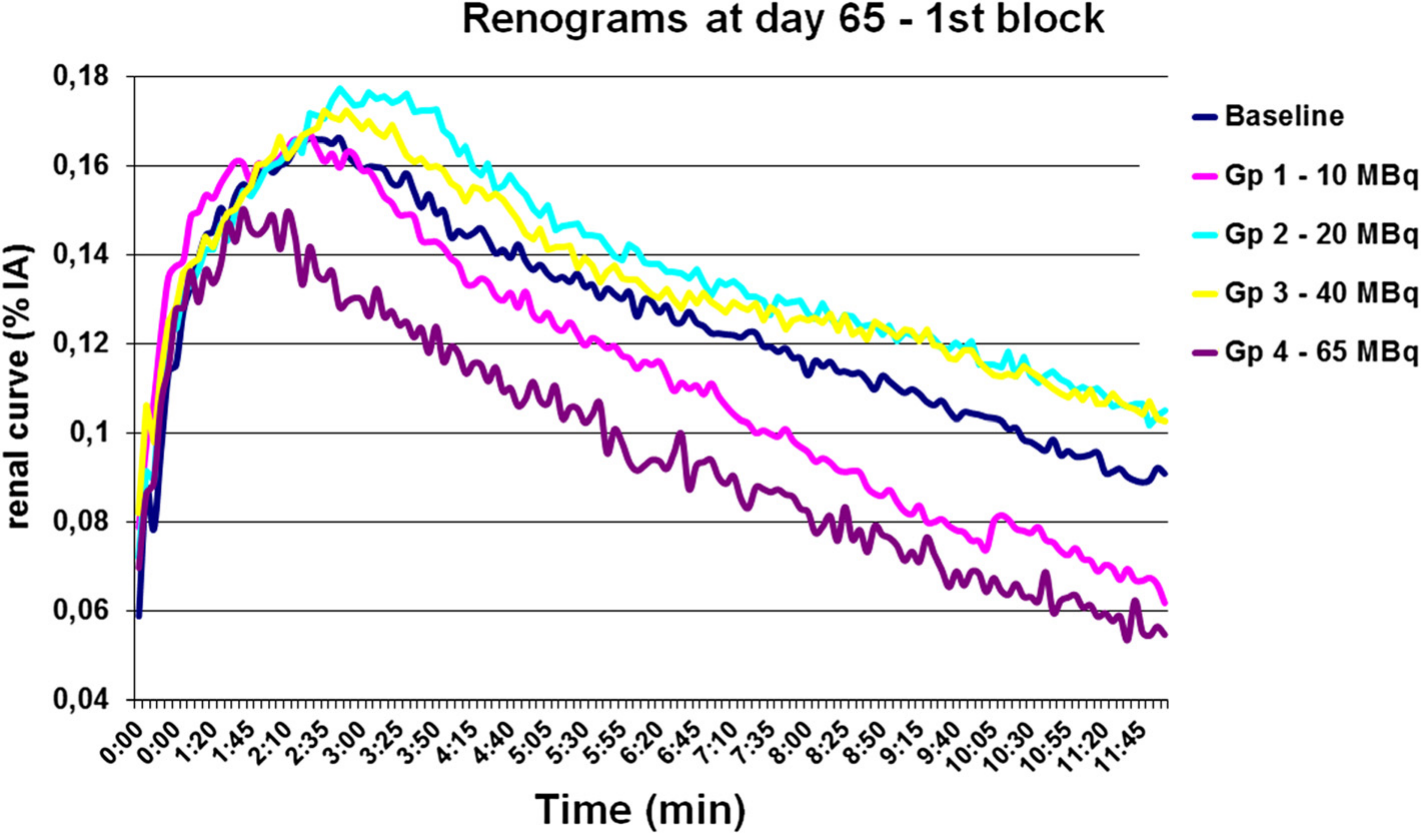
Renograms at follow-up examination at day 65. Renal dysfunction is reflected by decreased uptake and slope. Error bars not shown for clarity

Figure 2 illustrates the mean renograms for groups 1–4 at day 65. Relative to findings in the baseline group, the peak %IA amplitude was unchanged in group 1, increased in groups 2 and 3, and decreased in group 4, which had received the highest [177Lu]-DOTATATE activity. The time to peak %IA was likewise unchanged in group 1, delayed in groups 2 and 3, and shortened in group 4. The excretion capacity, assessed from the decline in %IA between peak and 10 min, was most impaired in group 3.

**Fig. 3:**
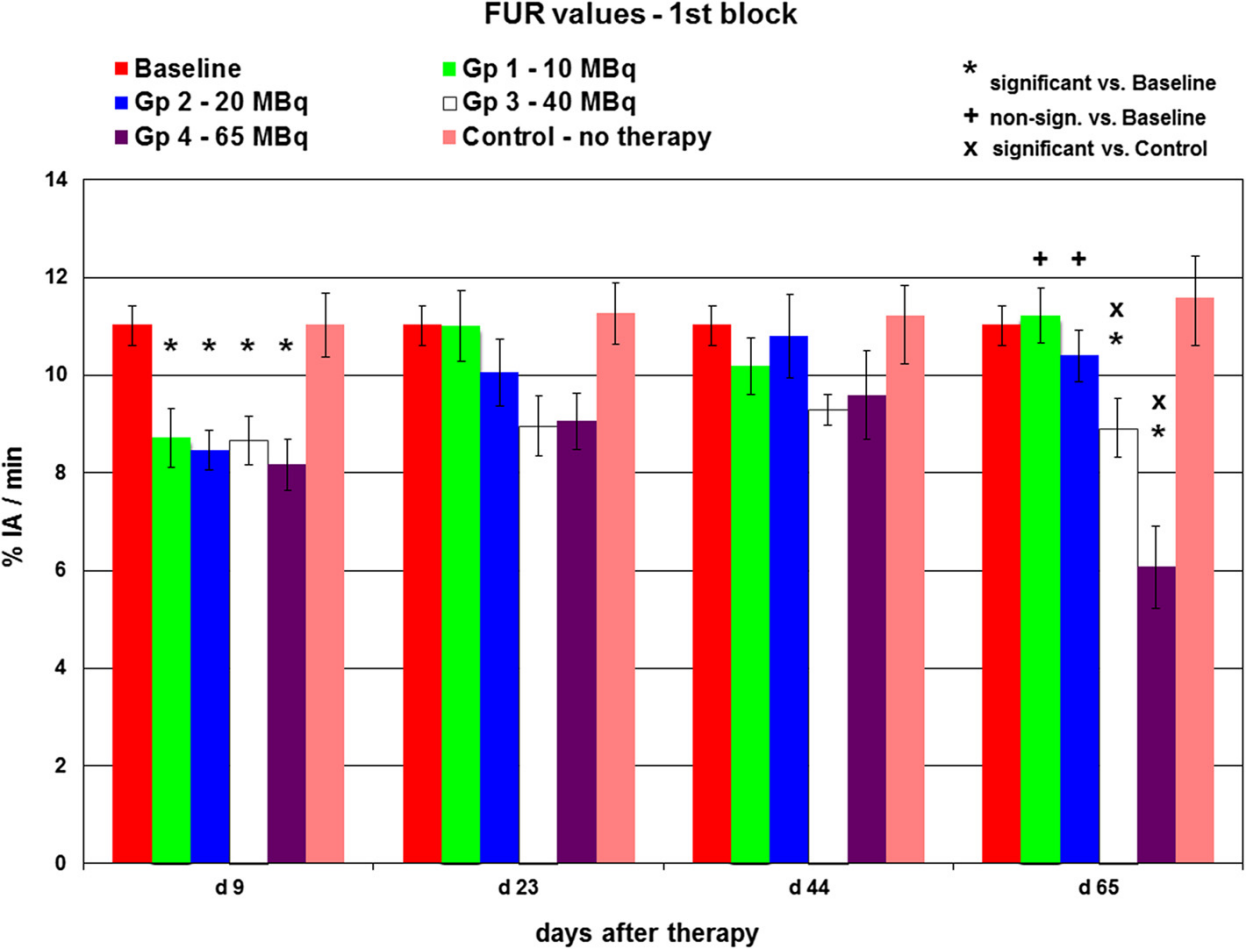
FUR values at baseline and days 9, 23, 44, and 65 in the first block of the study. All therapy groups exhibit decreased FUR at day 9. Increasing FUR values are obtained in the 10 and 20 MBq groups until day 65, whereas FUR in the 40 and 65 MBq groups remains significantly decreased

FUR values decreased significantly in all therapy groups at day 9 as compared to the baseline group (*p* < 0.01) (Fig. 3). During subsequent follow-up, FUR increased in group 1 (10 MBq), showing no significant difference at day 65 as compared to baseline values (11.2 ± 0.5 %IA/min vs. 11.0 ± 0.4 %IA/min). Similar findings were seen in group 2 (20 MBq). FUR values in groups 3 and 4 likewise recovered during initial follow-up but had significantly declined in a dose-dependent manner on the final scintigraphy day, when group 3 FUR was 8.9 ± 0.6 %IA/min (*p* < 0.05) and group 4 FUR was 6.0 ± 0.8 %IA/min (*p* < 0.01).

**Fig. 4:**
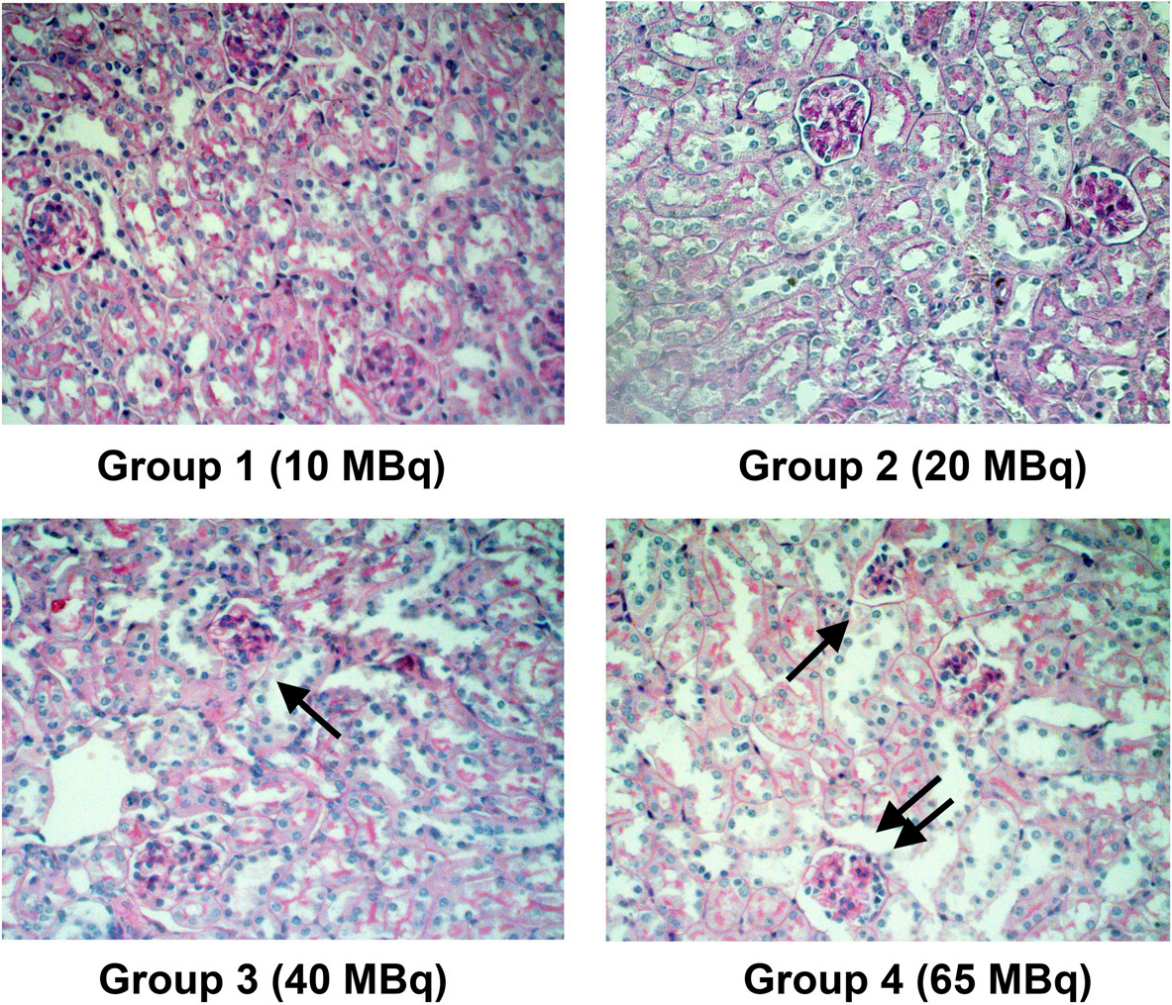
Histological sections (PAS) at day 65 in the first block of the study. With increasing therapy activities, renal damage gets more pronounced in terms of glomerular shrinkage (*arrow*), basal membrane thickening and tubular dilation (*double arrow*)

Histological sections at day 65 (Fig. 4) exhibited evidence for increasing renal damage (tubular dilatation and basal membrane thickening) with increasing PRRT activity. In particular, pronounced to severe renal injury in terms of glomeral shrinkage and tubular damage was most evident in the 40 and 65 MBq radiotherapy groups. However, no signs of pronounced necrosis were seen in any therapy group.

The synopsis of renogram, FUR analysis, and histopathological changes illustrated comprehensive renal damage at the two highest activities. We selected group 3 [177Lu]-DOTATATE activity (40 MBq) for the evaluation of pharmaceutical nephroprotection after enalapril treatment as a trade-off between producing a measureable defect and potentially excessive kidney failure.

### Evaluation of pharmaceutical nephroprotection

**Fig. 5:**
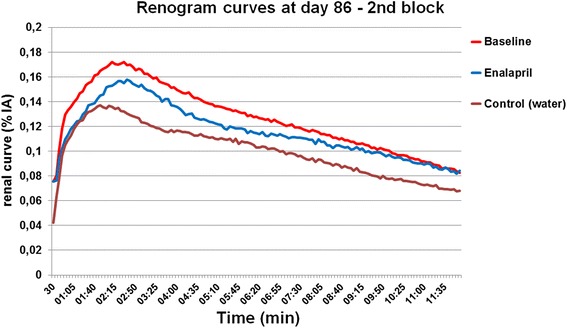
Renogram curves in the second part of the study at day 86. Both mean renogram curves in enalapril and control groups present a decreased peak value as compared to baseline, however, more pronounced in the control group. The flattening slopes indicate relatively higher renal damage in the water-treated control group as compared to the enalapril group. Regular slope in the enalapril group reveals normal renal excretion capacity

Mean renogram curves of enalapril-treated and control mice from day 9 after PRRT until day 86 are available as Additional file 1. Figure 5 shows the mean renogram curves of the enalapril and control groups at day 86 compared to baseline renograms prior to PRRT. The relatively elevated peaks in the mean renograms of the enalapril group and the steeper slopes relative to the control group are consistent with the preservation of renal function, although the curves are moderately impaired compared to baseline.

**Fig. 6:**
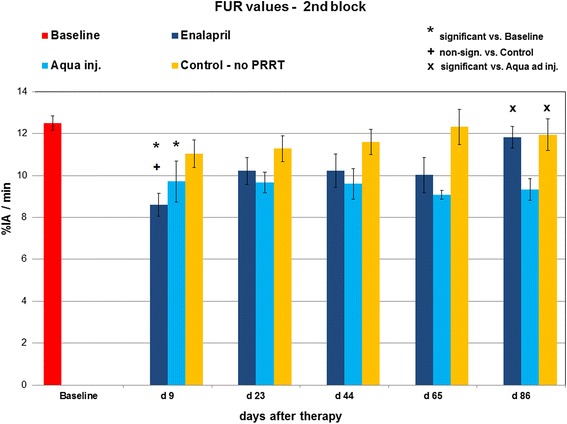
FUR values at baseline and days 9, 23, 44, 65, and 86 in the second block of the study. FUR remains relatively stable in the no PRRT control group. Decreased FUR values are presented in the PRRT groups a day 9. However, values in the enalapril group increase until day 86, whereas values in the water-treated control group remain significantly reduced as compared to baseline

We observed significantly decreased FUR values relative to baseline in both PRRT groups at day 9 (*p* < 0.01) (Fig. 6). At subsequent follow-up, FUR values tended to increase in the enalapril group, becoming non-significantly different from baseline (12.5 ± 0.3 %IA/min) at day 86 for enalapril treatment (11.8 ± 0.5 %IA/min), indicating rescue of renal function. In contrast, the mean FUR measurements in the control group remained significantly reduced relative to baseline at days 9, 23, 44, 65, and at day 86, when the FUR was 9.3 ± 0.5 %IA/min (*p* < 0.01).

**Fig. 7:**
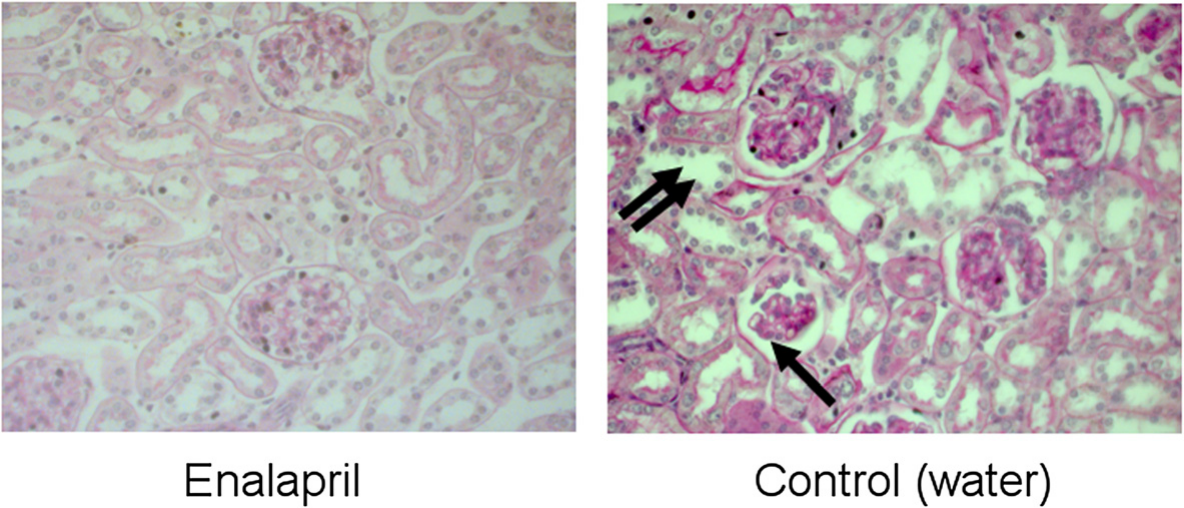
Histological sections (PAS) at day 86 in the second block of the study. Renal damage in terms of glomerular shrinkage (*arrow*), thickening of the basal membrane, and tubular dilation (*double arrow*) is presented in the control group (pronounced to severe renal damage). No signs of renal damage in the enalapril group

Histological sections at day 86, as seen in Fig. 7, exhibit renal damage manifesting in tubular dilatation, thickening of the basal membrane, and glomeral shrinkage in the control group (pronounced to severe damage). There are no signs of histological damage visible in enalapril-treated mice.

### Non-therapy control group

No relevant changes in mean renograms were observable during the follow-up period of 3 months (data not shown). Mean FUR values at the final day of follow-up revealed significant differences as compared to the water-treated control group, whereas no significant differences were measured in comparison to enalapril-treated mice underlining the nephroprotective effect of these treatment options (mean FUR after 3 months: 11.9 ± 0.8 %IA/min; *p* < 0.05 vs. water; *p* = n.s. vs. enalapril) (see Fig. 6). Histological sections did not reveal any renal damage in this group.

## Discussion

In clinical practice, the acceptable limit for kidney radiation during PRRT (23 Gy) is based upon findings with external beam radiation [33]. Mitigation of nephrotoxicity would offer the possibility to increase the radioactivity per cycle of PRRT and/or increase the number of cycles, to the benefit of NET patients. With this in mind, we investigated the nephroprotective characteristics of enalapril treatment in a mouse model of [177Lu]-DOTATATE therapy-induced nephropathy based upon encouraging results in studies based on external beam radiation [12, 24, 25].

Renal laboratory parameters such as blood urea nitrogen (BUN) and creatinine were unaffected in rats 6 weeks after PRRT, despite the occurrence of histological proven nephrotoxicity [34]. However, other studies are contradictory and describe an accordance of blood urea nitrogen and creatinine levels with kidney damage in mice, which was, however, true only in pronounced radiation-induced kidney damage [35, 36]. Therefore, we implemented MAG3 scintigraphy in order to quantify renal function in the course of treatments. MAG3 scintigraphy offers a non-invasive and individual evaluation of renal function in the course of radiation nephropathy [30]. Firstly, we determined the activity of [177Lu]-DOTATATE-induced nephrotoxic effects measured with MAG3 scintigraphy in mice. The establishment of a mouse model is of great importance, as the availability of diverse immune-deficient mouse models could deliver more insight into immunological processes of radiation nephropathy in prospective studies. Renograms proved to be highly variable in mice, such that we decided to test the FUR quantitative approach, which provided a more sensitive index of the extent of renal failure at day 65. The FUR approach also showed clearly the temporal dynamics of altered renal function, which included a transient decline at day 9 irrespective of the [177Lu]-DOTATATE activity, subsequent recovery at days 23 and 44, and the emergence of a clearly activity-dependent nephrotoxic effect at day 65. However, the transient decline evident at day 9 even in the present group of mice receiving only 10 MBq [177Lu]-DOTATATE suggests the occurrence of acute toxicity at an activity far below that required to provoke chronic renal failure, which takes several months to develop.

The superiority of FUR to renograms is substantiated by the findings of our group in a mouse model of ischemia-reperfusion injury [30]. We settled upon 40 MBq [177Lu]-DOTATATE, so as to obtain a distinct and reproducible impairment of renal function in the subsequent nephroprotection treatment arm of the study, without risking morbidity due to complete renal failure.

As noted above, the most common intervention for reducing kidney irradiation from radiolabeled somatostatin analogues is to competitively block their tubular reabsorption by co-infusion of basic amino-acids. Alternately, it might be possible to interfere with radiation-induced pathological processes leading to fibrosis and apoptosis. For example, the anti-oxidant amifostine attenuated renal damage in rats by the reduction of oxidative stress arising from [177Lu]-DOTATATE treatment [37]. Dual therapy with amino acids and anti-oxidants might have an additive or super-additive (synergistic) effect in rescuing renal function following internal irradiation, but this approach has yet to be investigated. Given that the RAAS pathway seems to play a central role in the pathophysiology of radiation-induced nephropathy [21], pharmaceutical intervention and modification of the RAAS should also present avenues for the mitigation of renal damage during PRRT. Enalapril is of proven efficacy in reducing injury in therapies leading to comparable patterns of renal damage as are provoked by [177Lu]-DOTATATE therapy. In particular, enalapril prevented radiation nephritis and fibrosis in rats after external beam radiation [24, 38]. The underlying mechanism of this effect of enalapril is uncertain; however, changes in glomerular capillary pressure and a reduction of circulating angiotensin II are discussed to be responsible for the mitigation of renal damage [24].

In our study, enalapril treatment likewise substantially rescued renal function, which was restored to baseline levels at day 86, as measured by FUR, and as also seen by renogram analysis. Furthermore, renal histology was entirely normal in the enalapril-treatment group, providing further support for the potential clinical use of enalapril in reducing functional and histopathological changes in the kidney after PRRT with [177Lu]-DOTATATE.

We consistently found that kidney function was reduced in the acute phase 1 week after [177Lu]-DOTATATE therapy, for the entire range of activities as depicted in significantly decreased FUR values. This seems to be the first such report after PRRT in mice. We did not obtain histological sections at day 9, and so we are unable to ascertain if this initial decline in renal function is associated with structural alterations. The functional impairment evoked by 40 MBq at day 9 was not reduced by enalapril treatment and persisted in the water-treated control group until day 86. However, mean FUR scores had normalized by day 86 in enalapril-treated mice. As data on pharmacological nephroprotection in mice after PRRT is still missing, the applied dose of enalapril was based upon results of previously published studies dealing with other scientific questions. A dose-response analysis would have been more accurate and will be performed in upcoming studies.

Functional data was based on MAG3 scintigraphy within a period of almost 3 months. A rat study has shown that MAG3 is a highly sensitive marker for the detection of renal damage after [177Lu]-DOTATATE therapy within a follow-up period of almost 4 months [39]. However, in a study dealing with folate-receptor-targeted therapy kidney damage was accurately measured using [99 m]-DMSA SPECT. This enabled an analysis of the correlation of kidney dose and kidney damage, which might offer an even more enhanced monitoring of kidney damage [35]. A previous study of our workgroup demonstrated that MAG3 scintigraphy with one single head of a triple-headed camera was accurate enough to depict renal function. However, there are several studies using a dedicated dynamic animal-SPECT [39, 40]. Studies comparing these different methods are missing, but, e. g., in terms of ROI vs. VOI analysis and thus accuracy SPECT is likely to be superior. Nevertheless, with the aid of renogram analysis and the calculation of the FUR, persistent impairment of renal function in mice after [177Lu]-DOTATATE therapy activity of at least 40 MBq is measurable by scintigraphy and was verified by histology.

## Conclusions

Treatment with enalapril for 3 months after radiotherapy with [177Lu]-DOTATATE mitigated renal damage in mice. Enalapril treatment could be an additional option for ameliorating the dose-limiting effects of radiation-induced kidney damage, thus allowing application of more effective therapy activities. Due to a moderate side effect profile and the applicability in patients with renal risk factors like hypertension and diabetes, which aggravate the nephrotoxicity of PRRT [41], enalapril treatment might be a promising therapy.

## Abbreviations

ACE, angiotensin-converting-enzyme; FUR, fractional uptake rate; HE, hematoxylin-eosin; MAG3, [99mTc]-mercaptoacetyltriglycine; NET, neuroendocrine tumor; PAS, periodic acid-Schiff reagent; PRRT, peptide receptor radionuclide therapy; RAAS, renin-angiotensin-aldosterone-system; ROI, region of interest

## Additional file

Additional file 1: Renogram curves for enalapril treatment in the second part of the study. (TIF 3031 kb)
