# Supplementary figures and images for: Nephroprotective effects of enalapril after [177Lu]-DOTATATE therapy using serial renal scintigraphies in a murine model of radiation-induced nephropathy
Source: EJNMMI Res. 2016 Aug 11;6:64. doi: 10.1186/s13550-016-0219-2 (PMC4980865; doi:10.1186/s13550-016-0219-2)

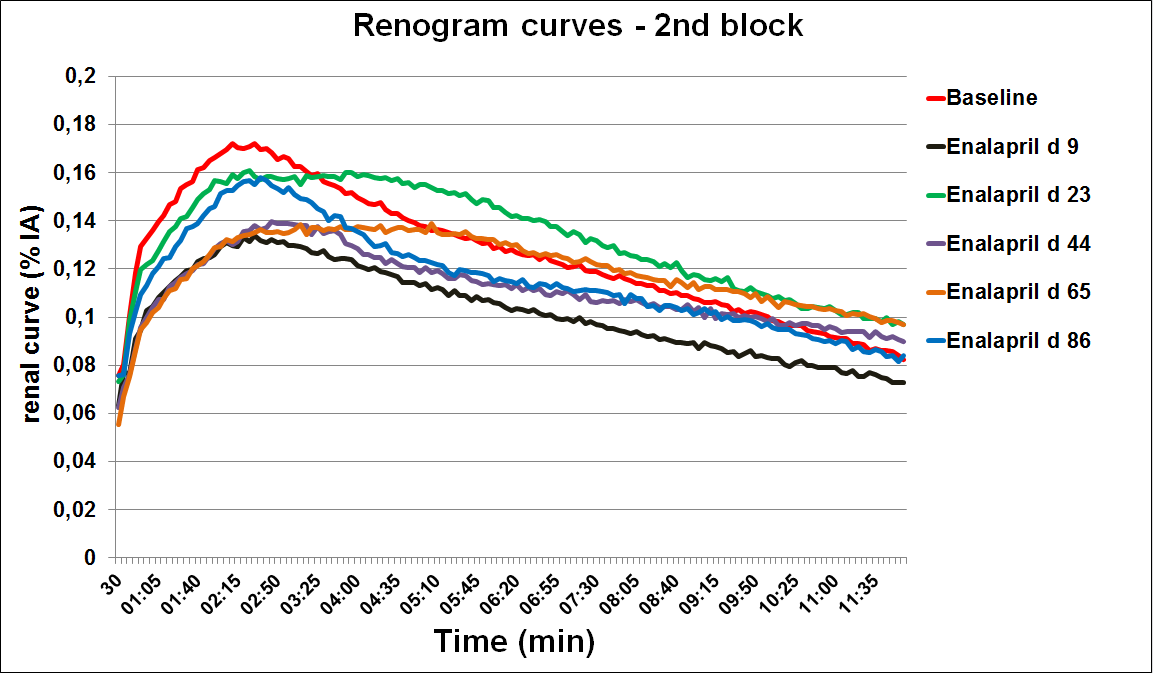

Supplement: Additional file 1: — Renogram curves for enalapril treatment in the second part of the study. (TIF 3031 kb) [file 13550_2016_219_MOESM1_ESM.tif]
